# Supplementary material for: Efficacy and Synergistic Potential of Cinnamon (Cinnamomum zeylanicum) and Clove (Syzygium aromaticum L. Merr. & Perry) Essential Oils to Control Food-Borne Pathogens in Fresh-Cut Fruits
Source: Antibiotics (Basel). 2024 Mar 31;13(4):319. doi: 10.3390/antibiotics13040319 (PMC11047545; doi:10.3390/antibiotics13040319)
Supplement: Supplementary file 1 [file antibiotics-13-00319-s001.zip › antibiotics-2897675-supplementary.pdf]

Article

Efficacy and synergistic potential of Cinnamon (*Cinnamomum zeylanicum*) and Clove (*Syzygium aromaticum* L. Merr. & Perry) essential oils to control food-borne pathogens in fresh-cut fruits

Ramona Iseppi [1], Eleonora Truzzi[2], Carla Sabia [1] and Patrizia Messi *[1]

[1] Department of Life Sciences, University of Modena and Reggio Emilia, Via G. Campi 287, 41125 Modena, Italy; ramona.iseppi@unimore.it (R.I.); carla.sabia@unimore.it (C.S.)

[2] Department of Chemical and Geological Sciences, University of Modena and Reggio Emilia, Via G. Campi 103, 41125 Modena, Italy; eleonora.truzzi@unimore.it (E.T.);

* Correspondence: patrizia.messi@unimore.it (P.M.)

Tel.: +39-059-205-5615 (P.M.)

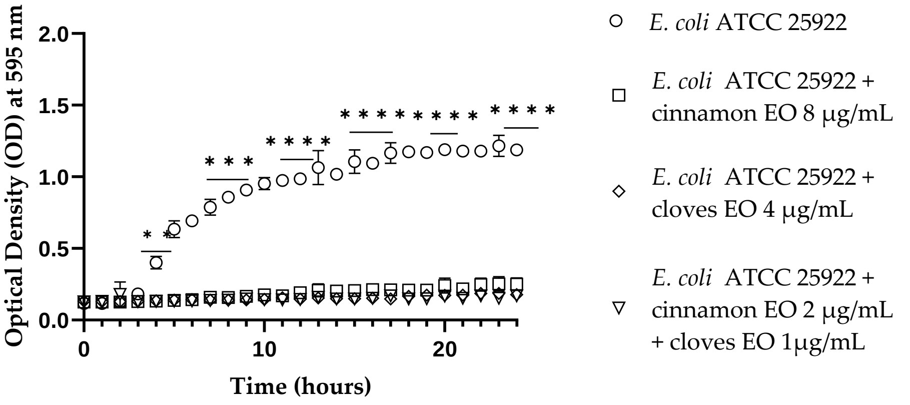

Figure S1: Time–kill studies of cinnamon and clove EOs alone and in combination against *Escherichia coli* ATCC 25922 viable cells. p-values of <0.05 (*), p <0.01 (**), p <0.001 (***) and p <0.0001 (****) were considered significant by t-test and ANOVA with Bonferroni correction. Results are expressed as mean ± SD of the three determinations (error bar = S.D.; n = 3).

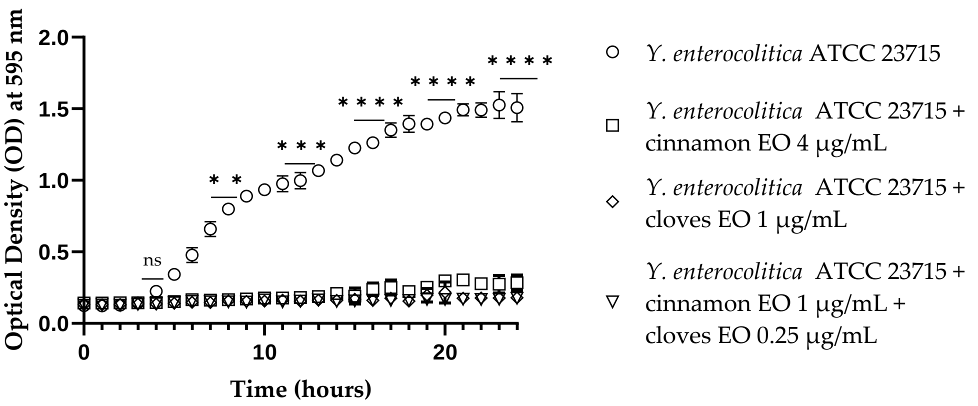

Figure S2: Time–kill studies of cinnamon and clove EOs alone and in combination against *Yersinia enterocolitica* ATCC 23715 viable cells. p-values of <0.05 (*), p <0.01 (**), p <0.001 (***) and p <0.0001 (****) were considered significant by t-test and ANOVA with Bonferroni correction. Results are expressed as mean ± SD of the three determinations (error bar = S.D.; n = 3).

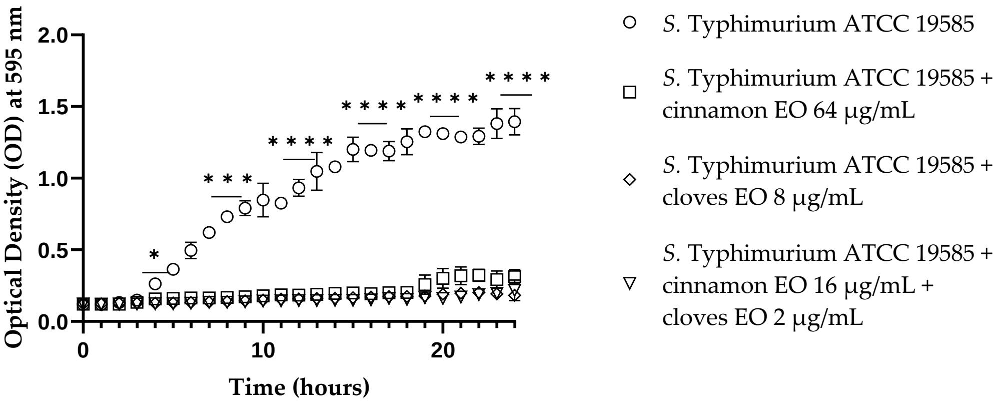

Figure S3: Time–kill studies of cinnamon and clove EOs alone and in combination against *Salmonella* Typhimurium ATCC 19585 viable cells. p-values of <0.05 (*), p <0.01 (**), p <0.001 (***) and p <0.0001 (****) were considered significant by t-test and ANOVA with Bonferroni correction. Results are expressed as mean ± SD of the three determinations (error bar = S.D.; n = 3).

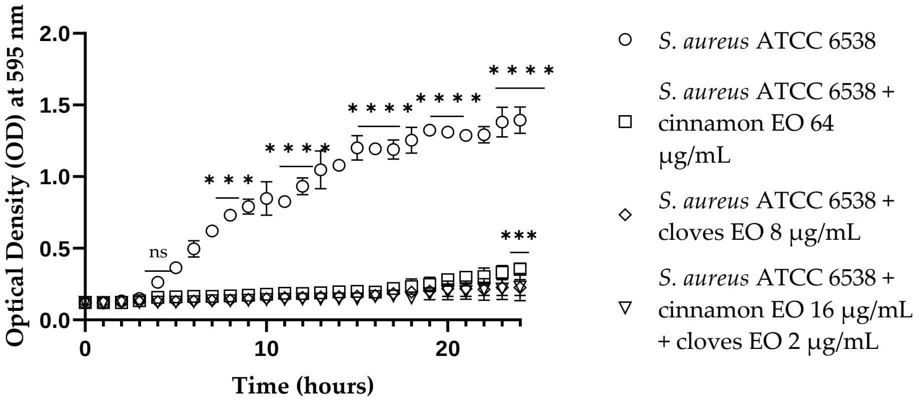

Figure S4: Time–kill studies of cinnamon and clove EOs alone and in combination against *Staphylococcus aureus* ATCC 6538 viable cells. p-values of <0.05 (*), p <0.01 (**), p <0.001 (***) and p <0.0001 (****) were considered significant by t-test and ANOVA with Bonferroni correction. Results are expressed as mean ± SD of the three determinations (error bar = S.D.; n = 3).
